# Legal Medicine and Otorhinolaryngology: Related Sciences

**DOI:** 10.1055/s-0044-1782198

**Published:** 2024-04-11

**Authors:** Ivan Dieb Miziara

**Affiliations:** 1 Full Professor of Legal Medicine, Bioethics, Social and Occupational Medicine, Faculty of Medicine, University of São Paulo, São Paulo, Brazil

Common questions in the corridors of courthouses, or among otorhinolaryngologists, are: “Is there any relationship between Otorhinolaryngology and Forensic Medicine?” and “To carry out a medical examination due to an otorhinolaryngological problem, is it necessary for the expert to be an otorhinolaryngologist?”. To try to answer these questions, we must first clarify the specialty of Legal Medicine and the concept of medical expertise.

There are more or less complete definitions of what Legal Medicine is, and one of them would be that of Odon Ramos Maranhão: “Legal Medicine is the science of applying medico-biological knowledge to the interests of constituted law and the law constituting”. 1 Concerning established law, the application of Legal Medicine occurs whenever the judicial authority seeks medical reports for the application of the law – be it criminal law, Civil law, or those listed by social security, administrative law, labor law etc. 2 In other words, whenever it is necessary to assess the severity of a bodily injury, the consequences caused by a personal injury, or the resulting disabilities in the social security and labor sphere, Forensic Medicine will be called upon to make its contribution.

The evolution of medical science, however, made Legal Medicine, like other specialties, interdisciplinary – as Oliver Schroeder Jr. well defined in 1974, training specialists to meet the common interests of Medicine and Law. 2 Due to this interdisciplinarity, Legal Medicine is today a multicurricular science with doctrinal foundations, as Flamínio Fávero 3 anticipated. Forensic Medicine, therefore, has its doctrine, which brings together all the different skills of other specialties into specific methods to meet the interests of Justice. Regarding this multidisciplinary knowledge, the parts related to Otorhinolaryngology are exemplary.

Those who work in Legal Medicine have expertise in human beings. Expertise is “seeing, hearing, examining, describing what you saw and examined, understanding, interpreting, and reporting” 4 to a judicial authority regarding a medical fact. After the examination has been completed, the expert informs the requesting authority through a medico-legal report. It is clear that when this medical fact involves an otorhinolaryngological issue, the Forensic Medicine expert must have sufficient knowledge of otorhinolaryngology.

If the specialist in Forensic Medicine is also a specialist in Otorhinolaryngology, his task will be easier. If this is not the case, their knowledge is insufficient, but if the situation is very complex, they can always consult a specialist in the field to obtain reliable information to help resolve the problem.

Concerning the various aforementioned fields of law, the intrinsic relationship between Forensic Medicine and Otorhinolaryngology is manifested in all of them – with benefits for otorhinolaryngology when using the common medico-legal methods. Another important aspect is when the otorhinolaryngologist faces legal proceedings for medical malpractice. To make a ruling on the case, the judge will have to resort to an expert examination that proves the existence of harm to the patient and the causal link between this harm and the medical act (in this case, an otorhinolaryngological act). The person who will carry out this examination to assist the judge (a layman in Medicine) in their conclusions is precisely the Forensic Medicine specialist.

## Legal Medicine, Otorhinolaryngology, and Criminal Law

In the field of Brazilian Criminal Law, issues involving otorhinolaryngological aspects, by way of illustration, fall under article 129 of the Brazilian Penal Code, which deals with bodily injuries. In this article, the legislator sought to divide bodily injuries into mild, severe, or very serious. 3 Suppose the injury suffered by the victim is of otorhinolaryngological origin (for example, a nasal fracture, traumatic hearing loss, or a change in body balance). In that case, the person who will quantify and qualify this injury will be a specialist in Forensic Medicine.

It is not always easy, as the medical expert makes an “interpretation of the law from a medical point of view”. 4 An injury sustained that results in an inability to perform one's usual occupations for more than 30 days is considered severe in the eyes of the law. However, how long will a nasal fracture take to heal for example? If it is more than 30 days, the injury will be considered severe; if it's less, take it. There is no consensus among otorhinolaryngologists on how long this fracture takes for complete repair. Several factors influence recovery time, such as gender, age, type of trauma, other associated fractures, need for surgical treatment etc. 5 6 The same happens in trauma or labyrinthine concussions, whose recovery time is unpredictable. 7

The relevance of Otorhinolaryngology in Criminal Law is ancient, given the frequency with which injuries to the facial mass occur—the description of maxillofacial trauma dates to Hippocrates (460–375 BC). In general, it tends to be the body region most frequently affected in otorhinolaryngological trauma (around 70% of the cases) because of social violence and, mainly, due to car accidents. 8

Therefore, the Forensic Medicine specialist must seek the best scientific evidence available to justify their conclusion under Criminal Law.

## Legal Medicine, Otorhinolaryngology, and Civil Law

When we talk about Civil Law from the perspective of Brazilian legislation, we talk about monetary compensation for pecuniary and non-pecuniary damages. We must understand the individual's body as their physical property – in terms of their dignity as a human being. According to the article 156 of the Brazilian Code of Civil Procedure, “the judge will be assisted by an expert when the proof of the fact depends on technical and scientific knowledge.” In cases in which the matter under discussion is in the medical field, “the legally qualified professional will be the doctor; among those registered to exercise this role, the doctor will need basic knowledge of Law and Legislation, the points that require technical analysis, the content of a medico-legal report, in short, a range of expertise that is the basis of the competence of the specialist in Legal Medicine”.

Civil disputes that demand otorhinolaryngological knowledge and financial compensation are generally those involving undesirable results of the otorhinolaryngologist's performance in the field of insurance of the most diverse origins. 9

Ziai et al. 10 went so far as to demonstrate that many disputes regarding malpractice in otorhinolaryngology in which surgeons were condemned occurred due to their work both in community service and in the private environment, but that the exercise of functions in educational institutions, as well as a high H index, there were many points in favor of the defendants.

Disputes regarding malpractice and medical negligence arise in all activities performed by otorhinolaryngologists, even in the most straightforward procedures such as tonsillectomies, which were the subject of a recent study covering the period from 1986 to 2020 in 24 American states; 11 when studying 42 cases, 6 patients suffered complications related to anesthesia, eleven had intraoperative bleeding, and fatal outcomes occurred in 14 cases.

## Legal Medicine, Otorhinolaryngology, and Labor and Social Security Law

Regarding labor, the intersection between Forensic Medicine and Otorhinolaryngology is one of the most common and traditional, mainly related to hearing loss and speech problems that arise in various fields of work. Here, the assessment of bodily harm resulting from work accidents or occupational illnesses is even more complex – including the need to establish an unequivocal causal link between the work activity and the damage caused –, due to the need to develop the degree of disability (permanent or temporary, partial, or total) produced by the sequelae. The expert in Forensic Medicine must have knowledge of the relevant legislation, specialization in Occupational Medicine, and understanding of the otorhinolaryngological signs and symptoms presented by the patients in the most common diseases linked to the performance of work – such as hearing loss induced by exposure to excessive noise and dysphonia of occupational origin –, as well as knowledge on how to perform assessments through audiometric exams, using decibel meters during workplace inspections, or using more specific exams such as laryngoscopy. Of course, it is always possible (and recommended) to resort to examinations performed by a specialist in the field to establish a diagnosis that does not raise doubts in the parties involved in the process.

## Final Considerations

As we have seen, the answers to the two questions asked initially to become simpler. The relationship between Forensic Medicine and Otorhinolaryngology is clear: they are related sciences that complement each other in all areas of law. Without a doubt, to carry out a good examination in the field of otorhinolaryngology, the recommended specialist practices Legal Medicine and has Medical Expertise and knowledge not only of the various medical specialties but also of the relevant legislation and medico-legal methods. It is necessary to assist Justice with competence and impartiality.
